# Supplementary material for: Effect of napping on a bean bag chair on sleep stage, muscle activity, and heart rate variability
Source: PeerJ. 2022 May 9;10:e13284. doi: 10.7717/peerj.13284 (PMC9097667; doi:10.7717/peerj.13284)
Supplement: Supplemental Information 1 — Abbreviations: REM, rapid eye movement; N2, non-REM sleep stage 2; N3, non-REM sleep stage 3; BC, bean bag chair; UC, urethane chair; d, effect size. Six participants exhibited N3 at each napping condition. REM sleep was excluded from the statistical comparison because only three participants manifested REM sleep during nap. Statistical significance was evaluated by the paired t-test. [file peerj-10-13284-s001.docx]

**Supplementary Information**

**Supplementary Table. Comparisons of quantitative electroencephalogram analyses during naps between bean bag and urethane chairs**

|  | Total | | | |
| --- | --- | --- | --- | --- |
|  | Delta | Theta | Alpha | Sigma |
| BC | 16.6 (2.5) | 12.6 (1.2) | 9.8 (1.1) | 6.3 (2.2) |
| UR | 17.1 (3.0) | 12.8 (1.6) | 9.6 (1.4) | 7.8 (1.3) |
| p | 0.205 | 0.575 | 0.611 | 0.082 |
| d | 0.165 | 0.130 | 0.143 | 1.102 |
|  | N2 | | | |
|  | Delta | Theta | Alpha | Sigma |
| BC | 19.0 (2.9) | 13.8 (1.3) | 10.4 (1.2) | 8.6 (1.6) |
| UR | 19.5 (3.9) | 13.9 (2.1) | 10.1 (1.6) | 8.3 (1.4) |
| p | 0.330 | 0.846 | 0.500 | 0.459 |
| d | 0.130 | 0.040 | 0.187 | 0.218 |
|  | N3 | | | |
|  | Delta | Theta | Alpha | Sigma |
| BC | 25.9 (6.3) | 15.5 (1.9) | 10.6 (1.6) | 10.5 (1.6) |
| UR | 25.9 (8.0) | 15.8 (3.2) | 11.0 (2.4) | 8.4 (1.6) |
| p | 0.129 | 0.220 | 0.373 | 0.052 |
| d | 0.002 | 0.103 | 0.163 | 1.354 |

Abbreviations: REM, rapid eye movement; N2, non-REM sleep stage 2; N3, non-REM sleep stage 3; BC, bean bag chair; UC, urethane chair; d, effect size.

Six participants exhibited N3 at each napping condition. REM sleep was excluded from the statistical comparison because only three participants manifested REM sleep during nap. Statistical significance was evaluated by the paired t-test.
